# Vacuum and Co-cultivation Agroinfiltration of (Germinated) Seeds Results in Tobacco Rattle Virus (TRV) Mediated Whole-Plant Virus-Induced Gene Silencing (VIGS) in Wheat and Maize

**DOI:** 10.3389/fpls.2017.00393

**Published:** 2017-03-22

**Authors:** Ju Zhang, Deshui Yu, Yi Zhang, Kun Liu, Kedong Xu, Fuli Zhang, Jian Wang, Guangxuan Tan, Xianhui Nie, Qiaohua Ji, Lu Zhao, Chengwei Li

**Affiliations:** ^1^Key Laboratory of Plant Genetics and Molecular Breeding, Zhoukou Normal University, ZhoukouChina; ^2^Henan Key Laboratory of Crop Molecular Breeding and Bioreactor, ZhoukouChina; ^3^College of Agronomy, Henan Agricultural University, ZhengzhouChina; ^4^College of Life Science and Agronomy, Zhoukou Normal University, ZhoukouChina; ^5^College of Life Science and Technology, Henan Institute of Science and Technology, XinxiangChina

**Keywords:** TRV-VIGS, vacuum and co-cultivation agroinfiltration, whole-plant level gene silencing, wheat, maize

## Abstract

Tobacco rattle virus (TRV)-mediated virus-induced gene silencing (VIGS) has been frequently used in dicots. Here we show that it can also be used in monocots, by presenting a system involving use of a novel infiltration solution (containing acetosyringone, cysteine, and Tween 20) that enables whole-plant level VIGS of (germinated) seeds in wheat and maize. Using the established system, phytoene desaturase (*PDS*) genes were successfully silenced, resulting in typical photo-bleaching symptoms in the leaves of treated wheat and maize. In addition, three wheat homoeoalleles of *MLO*, a key gene repressing defense responses to powdery mildew in wheat, were simultaneously silenced in susceptible wheat with this system, resulting in it becoming resistant to powdery mildew. The system has the advantages generally associated with TRV-mediated VIGS systems (e.g., high-efficiency, mild virus infection symptoms, and effectiveness in different organs). However, it also has the following further advantages: (germinated) seed-stage agroinfiltration; greater rapidity and convenience; whole-plant level gene silencing; adequately stable transformation; and suitability for studying functions of genes involved in seed germination and early plant development stages.

## Introduction

With fast development and wide applications of next-generation sequencing (NGS) technologies, genomes of increasing numbers of plants have been sequenced, including important crops such as barley, wheat, maize, cotton, and tomato (Schnable et al., 2009; Bräutigam and Gowik, 2010; Zhou et al., 2010; Brenchley et al., 2012; Kumar et al., 2012; Liu et al., 2012; Mayer et al., 2012; Paterson et al., 2012; The Tomato Genome Consortium, 2012; Jia et al., 2013; Ling et al., 2013). Hence, massive numbers of new genes have been identified, and functional analysis is required to decipher related biological traits and explore potential applications of these genes. A widely used approach in functional analysis is to generate mutations using chemical or physical agents (Kodym and Afza, 2003; Belfield et al., 2012; Hanafy and Mohamed, 2014; Serrat et al., 2014; Dhaliwal et al., 2015; Li et al., 2016; Zhang et al., 2016), T-DNA insertion (Weigel et al., 2000; Alonso et al., 2003), RNA interference (RNAi) (Kusaba, 2004; Mahmood-ur-Rahman et al., 2008; Wang et al., 2008, 2013), or genome editing (Shan et al., 2013; Wang et al., 2014; Kumar and Jain, 2015). However, those techniques often generate large numbers of mutants, and require tedious screening or genetic transformation procedures. In contrast, virus-induced gene silencing (VIGS) is a powerful tool for knocking-down genes in plants that is: quick and easy, does not require inefficient and cumbersome process genetic transformation, can overcome functional redundancy of gene families, avoids genotype-specific effects between different genetic backgrounds, timely target tissue-specific genes, suitable for large-scale functional analysis based on certain VIGS vectors, moreover it’s an effective system for analyzing genes which would normally be lethal once mutated (Dinesh-Kumar et al., 2003; Lu et al., 2003; Burch-Smith et al., 2004; George et al., 2010; Ramegowda et al., 2014).

As a technique based on post-transcriptional gene silencing (PTGS), it exploits plants’ natural defense systems that provide protection from invading viruses. Numerous virus genomes have been modified as VIGS vectors to date (Dinesh-Kumar et al., 2003; Lu et al., 2003; Unver and Budak, 2009; Becker and Lange, 2010; Senthil-Kumar and Mysore, 2011; Ramegowda et al., 2014). *Tobacco rattle virus* (TRV) could vigorously spread throughout the entire plant and show milder infection symptoms compared with other viruses, therefore, TRV-mediated VIGS (TRV-VIGS) is one of the most frequently used VIGS system for gene functional analysis(Burch-Smith et al., 2004). Moreover, it can silence genes in a broad range of plants, such as tomato, tobacco, *Arabidopsis*, rose, cotton, apple, and the parasitic plant *Striga hermonthica* (Liu et al., 2002a; Kirigia et al., 2014; Tian et al., 2014; Mustafa et al., 2016). TRV-VIGS in tobacco can be accomplished within 4 weeks, and is reportedly faster and easier than other available gene silencing techniques (George et al., 2012, 2015; Senthil-Kumar and Mysore, 2014). However, it is rarely reported that TRV-VIGS was used in monocot plant species, especially in wheat and maize. Barley stripe mosaic virus (BSMV)-mediated VIGS (BSMV-VIGS) is a useful tool for gene functional analysis in cereals that has been widely used in monocot plant research, but high-efficiency depended on microprojectile bombardment instruments or *in vitro* transcript or pre-infiltration of *Nicotiana benthamiana*, (Meng et al., 2009; Wang et al., 2011, 2015; Yuan et al., 2011; Chen et al., 2015; Jiao et al., 2015; Zhang et al., 2015; He et al., 2016) which limited its application. VIGS tools for maize is lacking, except the VIGS vectors derived from brome mosaic virus (BMV) (Ding et al., 2006) and recently reported virus vectors based on cucumber mosaic virus (CMV) strain ZMBJ-CMV and foxtail mosaic virus (FoMV) (Liu et al., 2016; Mei et al., 2016; Wang R. et al., 2016), however, the photo-bleaching symptom by silencing phytoene desaturase *(PDS)* gene is partial, suggesting that those VIGS systems in maize could not function at whole-plant level.

Bread wheat (*Triticum aestivum* L.), which has three closely related subgenomes (2*n* = 42; AABBDD), provides approximately 20% of all calories consumed by humans^1^. Due to the social and economic importance of this plant, there have been intensive efforts to characterize its genes and modify its traits to improve its yield, quality, and/or tolerance of biotic and abiotic stresses. As a hexaploid plant (with a genome of 17,000 megabases; about 40 times larger than rice genomes) wheat has at least three similar copies of most of its genes. Its high ploidy and repetitive DNA contents strongly hinder forward and reverse genetic analyses (Smith and Flavell, 1975; Choulet et al., 2010; Fitzgerald et al., 2010; Brenchley et al., 2012; Jia et al., 2013; Ling et al., 2013). Thus, technologies capable of targeting a specific copy or several homoeologous gene copies simultaneously would be highly beneficial for studying wheat and improving its agronomic traits. Maize (*Zea mays* L.) is another important calorie resource for both humans and livestock^2^. Unlike wheat, maize has a diploid genome, and it is widely used as a model plant in cereal research, hence there is abundant knowledge of maize genomes (~2000 Mb) and genetics, together with extensive associated resources and databases (Schnable et al., 2009). Moreover, both forward and reverse genetic techniques have been used in maize gene function analysis, such as gene overexpression, RNAi, and the popular genome editing tool CRISPR/Cas9 (McGinnis et al., 2007; Cho et al., 2014; Liang et al., 2014; Nannas and Dawe, 2015; Feng et al., 2016; Zhu et al., 2016). However, these methods require laborious, time-consuming, low-efficiency stable genetic transformation. Thus, there is a great need for new techniques that are quicker, more convenient, and more efficient for functional genomic analyses.

Here we report a new approach for applying TRV-VIGS to wheat, maize and potentially other plants, involving use of vacuum and co-cultivation agroinfiltration with a novel infiltration solution, which meets the needs for rapidity, convenience and highly efficient whole-plant level gene silencing.

## Materials and Methods

### Plant Materials and Powdery Mildew Fungus

Wheat cv. Xiaoyan 22 and maize cv. Zhengdan 958 were grown in a greenhouse under a photoperiod and thermal cycle of 16-h light (150 μmol.m^-2^.s^-1^) at 21°C and 8-h dark at 19°C with an average relative humidity of 55%. Following previously reported protocols (Cao et al., 2011; Wang et al., 2014), with minor modifications, virulent strains of *Blumeria graminis* f. sp. *tritici* (*Bgt*) isolate E18 were maintained on wheat cv. Jing411 plants, kept under the same photoperiod and thermal cycle, but at 70% relative humidity, in a growth chamber. The biological material used in this study is freely available for research purposes.

### Vector Construction

All oligonucleotide primers used (marked ZJXX) were synthesized by Sangon Biotech (Shanghai, China), and are listed in Supplementary Table S1. pTRV1 and pTRV2 VIGS vectors were constructed following a previously reported protocol (Liu et al., 2002b). For the construction of pTRV2-*SlPDS* a previously described 409-bp fragment of tomato *PDS* cDNA (Liu et al., 2002a) was amplified from tomato cDNA using PCR primers ZJ01and ZJ02. The PCR product was cloned into *Kpn*I-*Bam*HI-cut pTRV2 by *Kpn*I and *Bam*HI digestion and ligation. For the construction of pTRV2-*TaMLO* a 459-bp fragment derived from base 210–668 of wheat *MLO-B1* cDNA (Wang et al., 2014) was amplified from wheat cDNA using PCR primers ZJ11 and ZJ12. The PCR product was cloned into *Eco*RI-*Bam*HI-cut pTRV2 by *Eco*RI and *Bam*HI digestion and ligation.

### Vacuum Agroinfiltration and Co-cultivation

Wheat cv. Xiaoyan 22 and maize cv. Zhengdan 958 seeds were surface-sterilized by soaking in 75% (v/v) ethanol for 1 min and 2.5% sodium hypochlorite containing 0.1% Tween 20 for 6 min, then rinsed five times with sterile deionized water. The sterilized wheat seeds were placed in an incubator and germinated at 30°C for 30 h in the dark on 2–3 layers of filter paper soaked in distilled water. After this treatment, the emerging sprouts were about 3 mm long.

A 10 mL culture of each *Agrobacterium tumefaciens* strain to be used (see below) was grown overnight at 28°C in Luria–Bertani (LB) medium supplemented with 100 mg.L^-1^ of rifampicin and 50 mg.L^-1^of kanamycin. Then 200 μL of each overnight culture was inoculated into 20 mL portions of LB medium with antibiotics as above, and cultivated at 28°C until they had reached selected optical densities (OD_600_) of 0.04, 0.3, 0.5, 1.0, and 1.5.

**FIGURE 1:**
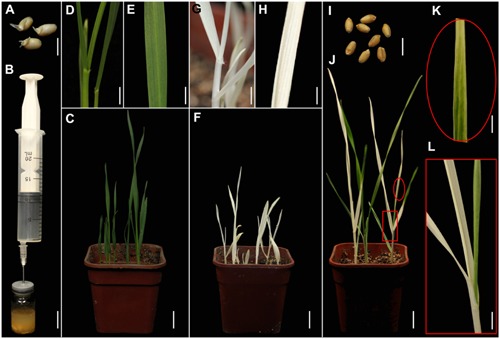
**TRV-mediated VIGS of the phytoene desaturase (*PDS*) gene in wheat through vacuum and co-cultivation agroinfiltration of (germinated) seeds with a novel agroinfiltration solution. (A)** Germinated wheat seeds used for agroinfiltration. **(B)** Vacuum agroinfiltration using a medical glass bottle sealed with a rubber plug and a 20-mL syringe. **(C–E)** Control plants grown from germinated wheat seeds at 16 days post-agroinfiltration (dpa) by *Agrobacterium tumefaciens* carrying pTRV2. **(D,E)** Show magnified details of a stem and leaf in **(C)**, respectively. **(F–H)** Treated plants grown from germinated wheat seeds at 16 dpa by *A. tumefaciens* carrying pTRV-*SlPDS* showing the photobleaching phenotype, **(G,H)** present magnified details of a stem and leaf in **(F)**, respectively. **(I)** Non-germinated wheat seeds used for agroinfiltration. **(J–L)** Treated plants grown from wheat seeds at 20 dpa by *A. tumefaciens* carrying pTRV-*SlPDS*, **(K,L)** present magnified images of a stem and leaf in **(J)**, respectively. Scale bars: **(A,I)**, 6.25 mm; **(B)**, 14.41 mm; **(D,G,L)**, 4.96 mm; **(E,H,K)**, 3.94 mm; **(C,F,J)**,14.9 mm.

**FIGURE 2:**
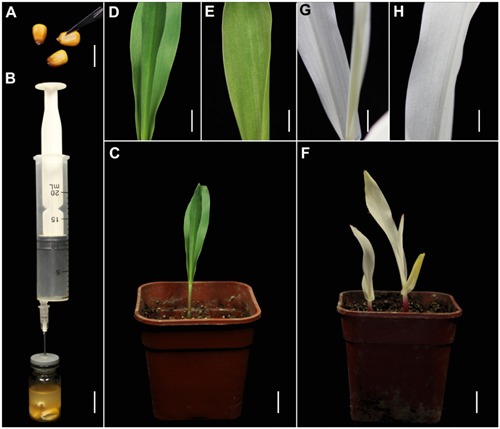
**TRV-mediated VIGS of the *PDS* gene in maize through vacuum and co-cultivation agroinfiltration of cut seeds with a novel agroinfiltration solution. (A)** Maize seeds cut above the embryo with a scalpel to enhance agroinfiltration. **(B)** Vacuum infiltration using the same equipment as shown in **Figure 1B**. **(C–E)** Control plants grown from cut maize seeds at 16 dpa by *A. tumefaciens* carrying pTRV2. **(F–H)** Treated plants grown from cut wheat seeds at 16 dpa by *A. tumefaciens* carrying pTRV-*SlPDS* showing the photobleaching phenotype, **(G,H)** present magnified images of a stem and leaf in **(F)**, respectively. Scale bars: **(A,B)**, 14.1 mm; **(C,F)**, 12.7 mm; **(D,E,G,H)**, 6.16 mm.

A 20 mL agroinfiltration liquid was made in a flask by mixing induced pTRV1 *A. tumefaciens* strain GV3101, respectively, with different induced *A. tumefaciens* strains GV3101 carrying different pTRV2 derived vectors (pTRV2, pTRV-*SlPDS*, pTRV-*MLO*) in 1:1 ratio, and supplemented with acetosyringone (AS) (19.62 mg.L^-1^), cysteine (Cys) (400 mg.L^-1^), and Tween 20 (5 ml.L^-1^). The sterilized (germinated) wheat and maize seeds were immersed in 5 mL agroinfiltration liquid in 10 mL medical glass bottles with rubber plugs (**Figures 1**, **2** and Supplementary Figure S3), shaken and subjected to ca. 20 kPa vacuum pressure generated using a 20-mL syringe for times ranging from 0 to 60 s. The resulting preparations were poured back into flasks and co-cultivated in a shaker at 28°C, 180 rpm, for a range of pre-selected co-cultivation durations (Supplementary Figure S3). After co-cultivation, the agroinfiltrated (germinated) seeds washed with sterilized water to remove surface-adsorbed Agrobacteria and grown in soil.

### Total RNA Extraction and RT-PCR

Total RNA was extracted from more than three independent biological replicates of leaves and roots of silenced and non-silenced plants (grown in soil, following the treatments mentioned above) until they were 16 days old using TRizol solution (Invitrogen, USA). First-strand cDNA was synthesized using mixtures containing 2 μg of total RNA, Oligo(dT)_18_, and M-MLV Reverse Transcriptase (Promega, USA) following Promega’s recommendations. The cDNAs were then used as templates for quantitative RT-PCR (qRT-PCR) with gene-specific primers outside the targeted region of genes for silencing, and PCR was performed with SYBR *Premix Ex Taq* (TaKaRa, Japan) and a CFX96 Real-Time System (Bio-Rad, USA) following the manufacturers’ recommendations. For detection of *TaPDS*, *ZmPDS*, *TaActin*, and *ZmActin*, primer pairs ZJ03/ZJ04, ZJ05/ZJ06, ZJ07/ZJ08, and ZJ09/ZJ10 (Supplementary Table S1), were used, respectively. Actin was used as an internal control.

### Powdery Mildew Infection and Microscopic Analyses

Powdery mildew infection and microscopic analyses were performed as previously reported (Hein et al., 2005; Shen et al., 2007) with some modifications. Leaves originating from the main shoot of sampled plants were collected and immediately placed in Petri dishes containing 1% (w/v) agar and 85 μM benzimidazole. The leaf segments were incubated at 21°C in continuous light (100 μmol.m^-2^.s^-1^) for 4 h, then inoculated with a virulent strain of *Bgt* E18 at a suitable density (˜150 conidiospores per cm^2^ of the inoculated leaves after inoculation). The fungus was allowed to grow on leaf segments for 60 h, then fixed with 1:1 (v/v) ethanol/acetic acid for 24 h, subsequently cleared with lactoglycerol (1:1:1 [v/v] lactic acid/glycerol/H_2_O) for 48 h, and stained for 7 s with 0.6% (w/v) Coomassie Brilliant Blue R250 in methanol (Sigma, USA) to visualize both the fungal structure and plant responses to attempted infection, then rinsed in distilled water and mounted in 50% (v/v) glycerol prior to microscopy. Samples were observed and analyzed under a BX41 light microscope (Olympus). More than 1,000 germinated conidiospores on each plant material used in every experiment were observed.

## Results

### Silencing of Wheat and Maize *PDS* Genes at Whole-Plant Level Using pTRV-*SlPDS* Vector

A *PDS* gene is frequently used to evaluate VIGS systems because leaves of *PDS*-silenced plants have easily recognizable photo-bleaching symptoms (Kumagai et al., 1995; Liu et al., 2002a,b). Furthermore, *PDS* genes in wheat, maize, and tomato are highly conserved, especially the fragment in the pTRV-*SlPDS* vector used here, where there is more than 75% identicality at the nucleotide level (Supplementary Figures S1, S2). It was previously shown that the *Nicotiana benthamiana PDS* gene (*NbPDS*) could be silenced by VIGS with TRV vectors carrying heterologous gene sequences of *NbPDS* (Senthil-Kumar et al., 2007). In this study, we constructed pTRV vectors according to a previous report (Liu et al., 2002a). TRV vectors (pTRV1, pTRV-*SlPDS*, pTRV2) were separately transformed into *A. tumefaciens* GV3101. A mixture of strains carrying pTRV1 and pTRV-*SlPDS* in a novel agroinfiltration solution including AS, Cys, and Tween 20 was used for *PDS* silencing, while a mixture of strains carrying pTRV1 and pTRV2 in the same agroinfiltration solution provided controls. Sterilized germinated wheat seeds (**Figure 1A**) or non-germinated wheat seeds (**Figure 1I**) were immersed in 5 mL portions of agroinfiltration solution with the *A. tumefaciens* culture mixtures and infiltrated by a simple vacuum infiltration method (**Figure 1B** and Supplementary Figure S3). Seeds that had already germinated when treated, and seeds that germinated after the agroinfiltration treatment, were further cultivated in the same agroinfiltration mixtures for selected times. After the seeds had been transferred to soil and cultivated in growth chambers for 16 days, systemic photo-bleaching was observed in all leaves of plants that developed from pTRV-*SlPDS*-inoculated germinated wheat seeds (**Figures 1F–H**). In contrast, partial photo-bleaching was observed in leaves of plants that developed from wheat seeds that were inoculated with pTRV-*SlPDS* before germination (**Figures 1J–L**), and control plants infected by pTRV2 developed normal leaves with no photo-bleaching symptoms under the same growth conditions (**Figures 1C–E**) These findings clearly show that *TaPDS* was specifically silenced in pTRV-*SlPDS* infected plants. qRT-PCR performed to check whether *PDS* was silenced in the whole plants (including roots and leaves), qRT-PCR data confirmed that *PDS* expression was knocked down in both roots and leaves of pTRV-*SlPDS* plants (Supplementary Figure S4). These observations demonstrate that the TRV-VIGS system silenced the targeted gene at the whole-plant level in wheat.

We also tested the suitability of this TRV-VIGS approach for maize, using the same vectors. Maize seeds are covered with a thick coat that may inhibit *Agrobacterium* infiltration, so we cut the coat of each seed above the embryo, using a scalpel, to improve the infection efficiency (**Figure 2A**). As in the wheat experiments, pTRV-*SlPDS* was used to silence *PDS* gene in maize plants (**Figure 2B**). Sixteen days after agroinfiltration, photo-bleaching symptoms were observed in all the leaves of plants infected by pTRV-*SlPDS* (**Figures 2F–H**), while control plants developed normal green leaves under the same conditions (**Figures 2C–E**), suggesting that *ZmPDS* was successfully silenced by pTRV-*SlPDS*. qRT-PCR confirmed that PDS expression was knocked down in both the roots and leaves of pTRV-*SlPDS*-infected maize plants (Supplementary Figure S4), showing that the developed TRV-VIGS system can silence *PDS* genes in maize at the whole-plant level.

### Optimization of TRV-VIGS in Wheat and Maize

**FIGURE 3:**
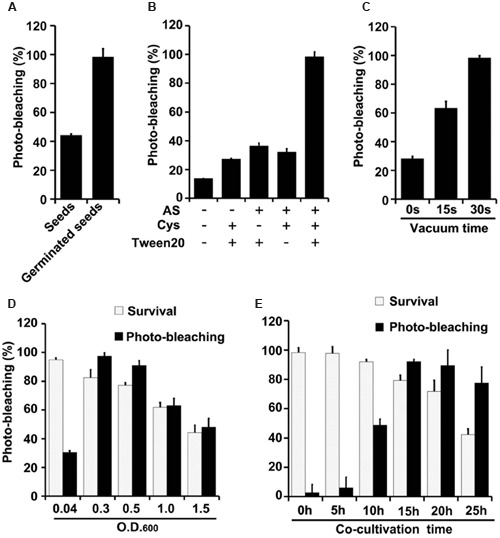
**Optimization of factors influencing results of the newly developed TRV-mediated VIGS in wheat. (A)** Silencing efficiency is significantly higher in plants that develop from pTRV-*SlPDS*-inoculated germinated wheat seeds than in plants that develop from wheat seeds treated before germination. **(B)** Effects of indicated components of the agroinfiltration liquid on silencing efficiency. **(C)** Effects of varying vacuum-induced agroinfiltration times on silencing efficiency. **(D)** Effects of the density (OD_600_) of the *A. tumefaciens* culture used in the agroinfiltration on silencing efficiency. **(E)** Effects of varying the co-cultivation time on silencing efficiency and survival rates of wheat plants. Values are means ± SD obtained from at least three independent experiments. Note: Optimal conditions of the other factors were used when optimizing the presented parameter.

**FIGURE 4:**
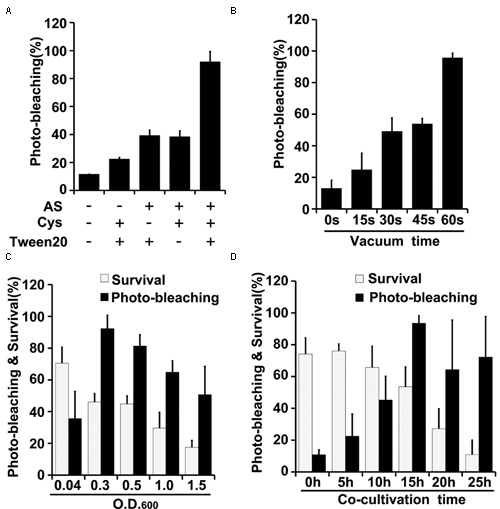
**Optimization of factors influencing results of the newly developed TRV-mediated VIGS in maize. (A)** Effects of indicated components of the agroinfiltration liquid on silencing efficiency. **(B)** Effects of varying vacuum-induced agroinfiltration on silencing efficiency. **(C)** Effects of the density (OD_600_) of the *A. tumefaciens* culture used in the agroinfiltration on silencing efficiency. **(D)** Effects of varying the co-cultivation time on silencing efficiency and survival rates of wheat plants. Values are means ± SD obtained from at least three independent experiments. Note: Optimal conditions of the other factors were used when optimizing the presented parameter.

Effects of varying several parameters (agroinfiltration solution components, plant materials used for infiltration, co-cultivation time, vacuum infiltration time, and *Agrobacterium* density) were studied to optimize the TRV-VIGS system in wheat and maize (**Figures 3**, **4**). The gene silencing efficiency was assessed by calculating percentages of the plants that showed photobleaching symptoms in each experiment. The silencing efficiency was twice as high when germinated wheat seeds were infiltrated than when viable but non-geminated seeds were used (**Figure 3A**). In addition, all components of the infiltration solution (AS, Cys, and Tween 20), based on solutions used in our previous studies (Xu et al., 2014; Zhang et al., 2014), with modifications, were needed for high silencing efficiency. Omission of any one of them reduced the silencing efficiency two- to threefold in both wheat (**Figure 3B**) and maize (**Figure 4A**).

Vacuum agroinfiltration has been used in VIGS systems in various previous studies (Tague and Mantis, 2006; George et al., 2010, 2012, 2015; Kirigia et al., 2014; Tian et al., 2014). In the experiments reported here it was applied using 20 mL syringes and 10 mL medical glass bottles (**Figures 1B**, **2B** and Supplementary Figure S3). With this approach, agroinfiltration with suction for 30 s, suction for 15 s, and no suction resulted in silencing efficiencies of ca. 97, 63, and 28%, respectively, in wheat (**Figure 3C**). The optimal time for maize TRV-VIGS was longer, as vacuum agroinfiltration for 60, 45, 30, 15, and 0 s resulted in silencing efficiencies of ca. 92, 54, 49, 25, and 13%, respectively (**Figure 4B**).

Tests in which the density of the *Agrobacterium* used in vacuum and co-cultivation agroinfiltration was varied indicated that cultures with an OD_600_ of 0.3 were optimal, providing higher silencing efficiencies and/or plant survival rates, in experiments with both wheat and maize, than cultures with an OD_600_ of 0.5, 1.0 or 1.5 (**Figures 3D**, **4C**). However, maize survival rates were much lower than those of wheat after agroinfiltration with *Agrobacterium* at an of OD_600_ = 0.3 (46 and 82%, respectively; **Figures 3D**, **4C**). Finally, the co-cultivation duration after agroinfiltration also influenced silencing efficiencies and plant survival rates, which were highest (for both wheat and maize) with a co-cultivation duration of 15 h (**Figures 3E**, **4D**)

Thus, the optimized protocol (giving almost 100% silencing efficiency in wheat and maize) involved agroinfiltration of germinated wheat seeds and cut maize seeds by vacuuming for 30 and 60 s, respectively, followed by co-cultivation for 15 h with *Agrobacterium* at OD_600_ = 0.3 in the all-component agroinfiltration solution. During the optimization experiments, symptoms like photo-bleaching were observed in several control plants agroinfiltrated with *Agrobacterium* at high concentrations when vacuum infiltration and co-cultivation periods were long. However, frequencies of these symptoms were significantly lower than among the plants subjected to the silencing treatment. These unexpected symptoms might have been due to effects of excessive *Agrobacterium* infection on the control plants.

### Simultaneous Silencing of Three *MLO* Homoeoalleles in Hexaploid Wheat Confers Resistance to Powdery Mildew

*MLO* genes encode proteins that repress defenses against powdery mildew diseases (Buschges et al., 1997; Acevedo-Garcia et al., 2014). Accordingly, mutation or silencing of *MLO* alleles can lead to broad-spectrum and durable resistance to powdery mildew caused by various fungal pathogens in barley, wheat, tomato, and cotton (Piffanelli et al., 2004; Bai et al., 2008; Varallyay et al., 2012; Acevedo-Garcia et al., 2014; Wang et al., 2014; Wang X. et al., 2016). Powdery mildew caused by *B. graminis* f. sp. *tritici* (*Bgt*), one of the most destructive wheat pathogens globally, can reduce wheat yields by 20–40%. As a hexaploid plant, wheat has three subgenomes, there are more than three copies of *MLO* genes, with functional redundancy, in wheat genomes. Wang et al. (2014) found that knocking out three highly conserved *MLO* homoeoalleles from different wheat subgenomes (*TaMLO-A1*, *TaMLO-B1*, and *TaMLO-D1*; which have about 98 and 99% identicality at the nucleotide and protein levels, respectively) by genome editing led to broad-spectrum and durable resistance to *Bgt*. Thus, to further test our TRV-VIGS system, these three wheat *MLO* homoeoalleles were silenced, using cDNA of a highly conserved 459 bp fragment of the *MLO* homoeoallele from wheat subgenome B (*MLO-B1*), which is about 98% identical to the other two *MLO* homoeoalleles, from wheat subgenomes A and D, as the target sequence for VIGS vector construction (Supplementary Figure S5).

**FIGURE 5:**
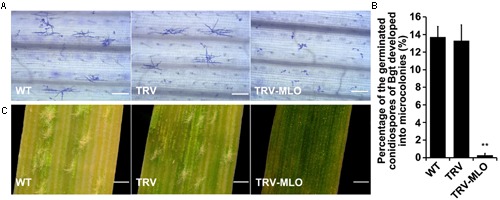
**TRV-mediated VIGS of *TaMLO* confers resistance to powdery mildew in wheat. (A)** Micrographs showing microcolony formation of *Blumeria graminis* f. sp. *tritici* (*Bgt*) virulent isolate E18 on surfaces of leaves of wild-type (WT) plants, VIGS control plants infected by *A. tumefaciens* carrying pTRV2 (TRV) and *TaMLO*-silenced plants infected by *A. tumefaciens* carrying pTRV-*MLO* (TRV-MLO), 60 hours post-inoculation (hpi) with *Bgt* virulent isolate E18. Powdery mildew spores and colonies were stained with Coomassie Brilliant Blue; scale bars, 0.2 mm. **(B)** Percentages of germinated *Bgt* conidiospores that developed into microcolonies on leaves of WT, TRV, and TRV-MLO plants at 60 hpi with *Bgt* virulent isolate E18; more than 1,000 germinated conidiospores on each plant material in every experiment were observed. Values are means ± SD obtained from at least three independent experiments, **P < 0.01 (*t*-test). **(C)** Macroscopic phenotypes of *Bgt* infection on leaves from WT, TRV, and TRV-MLO plants at 7 days post-inoculation (dpi) with *Bgt* virulent isolate E18; scale bars, 0.82 mm.

Sixteen days after agroinfiltration, leaves from the treated plants, and controls, were sampled and placed on 1% agar plates containing 85 μM benzimidazole, incubated for at least 4 h then inoculated with *Bgt* conidiospores. The leaves were stained 60 hours post-inoculation (hpi) for microscopic observation. Percentages of germinated conidiospores that developed into microcolonies were similar in pTRV plants and non-agroinfiltrated plants inoculated with *Bgt*, and significantly higher (in both cases) than in *MLO*-silenced (pTRV*-MLO*) plants inoculated with *Bgt* (*P* < 0.01) (**Figures 5A,B**). Moreover, clear mycelial colonies were macroscopically observed on leaves of the control plants 7 days post-inoculation (7 dpi), while very few were found on leaves of pTRV*-MLO* plants (**Figure 5C**), in accordance with the microscopy results. As *TaMLO-A1*, *TaMLO-B1*, and *TaMLO-D1* are functionally redundant (Wang et al., 2014), silencing one or two of the three *MLO* homoeoalleles in wheat cannot eliminate wheat susceptibility to powdery mildew caused by *Bgt*. Thus, these findings indirectly indicate that all three *MLO* homoeoalleles were simultaneously silenced in the pTRV*-MLO* plants generated using the developed TRV-VIGS system, since they showed high resistance to powdery mildew caused by *Bgt*.

## Discussion

Functional analysis of genes identified in plant genome sequencing projects is a major challenge in post-genomic study. VIGS is one of the most widely applied techniques in such analysis because it is quick, convenient, easy to scale for high-throughput analyses, avoids needs for genetic transformation, and suitable for targeting redundant genes and genes required for plant survival (Dinesh-Kumar et al., 2003; Lu et al., 2003; Burch-Smith et al., 2004; George et al., 2010; Ramegowda et al., 2014). Nearly 40 kinds of viruses have been modified for use as VIGS vectors, mostly RNA viruses (Senthil-Kumar and Mysore, 2011; Ramegowda et al., 2014; Wang R. et al., 2016). TRV is particularly suitable for already mentioned reasons, and it has been used in VIGS to silence genes in many dicot plans, but has rarely (if ever) been applied in monocot plants (Liu et al., 2002a; Tian et al., 2014; Shen et al., 2015).

Wheat and maize are among the most important cereal crops globally, so an efficient VIGS tool for functional analysis of genes in these plants would be highly valuable. VIGS systems based on BSMV vectors that can silence genes in barley and wheat have been developed, but as mentioned in the section “Introduction” they have several limitations (Holzberg et al., 2002; Bruun-Rasmussen et al., 2007; Meng et al., 2009; Yuan et al., 2011; Chen et al., 2015; Jiao et al., 2015; Wang et al., 2015; Zhang et al., 2015; He et al., 2016). Moreover, the only VIGS tools for maize do not function at the whole-plant level, because they only lead to partial photo-bleaching when they are used to silence *PDS* in maize leaves (Ding et al., 2006; Liu et al., 2016; Mei et al., 2016; Wang R. et al., 2016). This report demonstrates that TRV-VIGS, previously used for silencing dicot genes, can also silence genes in monocots. Moreover, the presented TRV-VIGS system is highly efficient, rapid, convenient, and suitable for silencing genes (at the whole-plant level) in wheat, maize, and possibly other monocots. Thus, conversely to BSMV-derived vectors, which can be applied in dicots (*N. benthamiana*) as well as monocots (Yuan et al., 2011), TRV-derived vectors can be used for VIGS in monocots as well as dicots. The findings clearly show that vectors derived from various viruses can be used for VIGS in non-host plants, even plants with large phylogenetic distances from the viruses’ hosts. The apparent lack of suitable VIGS vectors for many plant species has hindered widespread adoption of VIGS (Scofield and Nelson, 2009; Senthil-Kumar and Mysore, 2011), but our results and previous reports clearly show that it can be extended to recalcitrant plants by using currently available vectors (Yuan et al., 2011).

Phytoene desaturase (*PDS*) genes have been commonly used as reporter genes in VIGS systems because silencing them results in easily-detected photo-bleaching symptoms (Kumagai et al., 1995). Thus, we used pTRV-*SlPDS* (which targets nucleotide sequences that are highly conserved in wheat, maize, and tomato *PDS* genes) to silence *PDS* genes in wheat and maize (Supplementary Figures S1, S2). Wheat *TaPDS* and maize *ZmPDS*, which share 76.53 and 78.24% identity with *SlPDS*, respectively (Supplementary Figures S1, S2), were successfully silenced by pTRV-*SlPDS*-mediated VIGS with the developed system, in accordance with published recommendations of a 75% sequence identity threshold for VIGS (Holzberg et al., 2002).

Vacuum-aided agroinfiltration has proven ability to increase infection efficiency in genetic transformation and VIGS procedures, especially for plants that have high resistance to infiltration (Tague and Mantis, 2006; George et al., 2010, 2012, 2015; King et al., 2014; Tian et al., 2014). Thus, we employed vacuum-aided agroinfiltration and found that applying it for 30 and 60 s resulted in the highest silencing efficiencies for wheat and maize, respectively. Co-cultivation of (germinated) seeds with *Agrobacterium*, which might increase the percentage of infected plant cells, was first applied to increase the silencing efficiency of VIGS. The results showed that there is a clear optimum *Agrobacterium* density for efficient VIGS. Moreover, extending the duration of co-cultivation beyond a threshold time reduced the VIGS efficiency, possibly because excessive infection by *Agrobacterium* reduces plant survival rates (**Figures 3E**, **4D**).

Other factors known to affect agroinfiltration results include the plant materials used, the density of bacteria, and components in the agroinfiltration solution (Xu et al., 2014). Suitable plant materials are also important for successful VIGS, as shown here. The developed VIGS system could be applied to both germinated and un-germinated wheat seeds, but the VIGS efficiency and extent of photo-bleaching symptoms were higher in plants that developed from germinated seeds (**Figures 1**, **3A**), suggesting that germinated wheat seeds were much less resistant to agroinfiltration than un-germinated seeds. However, agroinfiltration of un-germinated seeds with cut coats resulted in the highest VIGS efficiency and extent of photo-bleaching symptoms in maize. Thus, seeds of different plant species probably have different optimum stages for VIGS agroinfiltration because of differences in their anatomy, morphology, and (perhaps) physiology.

The optimal OD_600_ of Agrobacterium for agroinfiltration, among those tested, was found to be 0.3. Lower concentrations resulted in lower VIGS efficiency, while higher concentrations impaired plant survival (**Figures 3D**, **4C**). Thus, the *Agrobacterium* density should be carefully optimized in terms of both of these variables to optimize VIGS results. Confirming the importance of the infiltration liquid’s composition (Xu et al., 2014), all three components (AS, Cys, and Tween 20) in our agroinfiltration solution played important roles in the TRV-VIGS of both wheat and maize, as omitting any of them significantly reduced the silencing efficiency (**Figures 3B**, **4A**). This suggests that they have synergistic effects in agroinfiltration.

As previously mentioned, resistance to powdery mildew has been generated in hexaploid bread wheat by simultaneous editing of three *MLO* homoeoalleles, but the transformation was inefficient, tedious, and laborious (Wang et al., 2014). Our novel TRV-based VIGS system can conveniently and simultaneously silence all three *MLO* homoeoalleles in wheat by targeting a highly conserved fragment, and results in similar resistance to powdery mildew. Moreover, whole-plant level silencing is difficult using conventional VIGS methods, in which leaves are often inoculated by agroinfiltration, thereby preventing functional analysis of some genes, especially those involved in plants’ early development. In contrast, macroscopic observation (**Figures 1**, **2**) and qRT-PCR analysis (Supplementary Figure S4) showed that the developed TRV-VIGS system, using (germinated) seeds, resulted in whole-plant gene silencing, which will extend the range of genes that can be subjected to VIGS. The findings also suggest that agroinfiltration in early growth stages of plants could increase the extent of VIGS-induced symptoms.

In summary, the developed TRV-VIGS system enables rapid, convenient, highly efficient whole-plant level silencing of monocot genes, without complex transformation manipulation. Thus, it appears to have high potential utility in functional analyses of monocot genes.

## Author Contributions

JZ and CL conceived the original screening and research plans; JZ and DY supervised the experiments; JZ, DY, XN, QJ, and LZ performed most of the experiments; YZ, KL, KX, FZ, JW, and GT provided technical assistance to JZ, DY, XN, QJ, and LZ; JZ and DY designed the experiments and analyzed the data; JZ, DY, and CL conceived the project and wrote the article with contributions from all the authors; CL supervised and complemented the writing.

## Conflict of Interest Statement

The authors declare that the research was conducted in the absence of any commercial or financial relationships that could be construed as a potential conflict of interest.

## Footnotes

1 http://faostat.fao.org/site/339/default.aspx

2 http://faostat.fao.org
