# Supplementary material for: Vacuum and Co-cultivation Agroinfiltration of (Germinated) Seeds Results in Tobacco Rattle Virus (TRV) Mediated Whole-Plant Virus-Induced Gene Silencing (VIGS) in Wheat and Maize
Source: Front Plant Sci. 2017 Mar 22;8:393. doi: 10.3389/fpls.2017.00393 (PMC5360694; doi:10.3389/fpls.2017.00393)
Supplement: Supplementary file 1 [file Data_Sheet_1.pdf]

Supplementary Material

• Vacuum and co-cultivation agroinfiltration of (germinated) seeds results in tobacco rattle virus (TRV) mediated whole-plant virus induced gene silencing (VIGS) in wheat and maize

Ju Zhang[1,2,#], Deshui Yu[1,2,#], Yi Zhang[1,2,3], Kun Liu[1,2], Kedong Xu[1,2], Fuli Zhang[4], Jian Wang[1,2], Guangxuan Tan[1,2], Xianhui Nie[1,2,4], Qiaohua Ji[1,2,4], Lu Zhao[1,2,4], Chengwei Li[1,2,5*]

[1] Key Laboratory of Plant Genetics and Molecular Breeding, Zhoukou Normal University, Zhoukou, 466001, China;

[2] Henan Key Laboratory of Crop Molecular Breeding & **Bioreactor**, Zhoukou, 466001, China;

[3] College of Agronomy, Henan Agricultural University, Zhengzhou, 450002, China;

[4]College of Life Science and Agronomy, Zhoukou Normal University, Zhoukou, 466001, China;

[5] College of Life Science and Technology, Henan Institute of Science and Technology, Xinxiang, 453003, China.

# These authors contributed equally to this study.

*** Correspondence:**
Correspondence and requests for materials should be addressed to C.-W. L (lichengweiwau@hotmail.com)

1 Supplementary Figures and Tables

1.1 Supplementary Figures

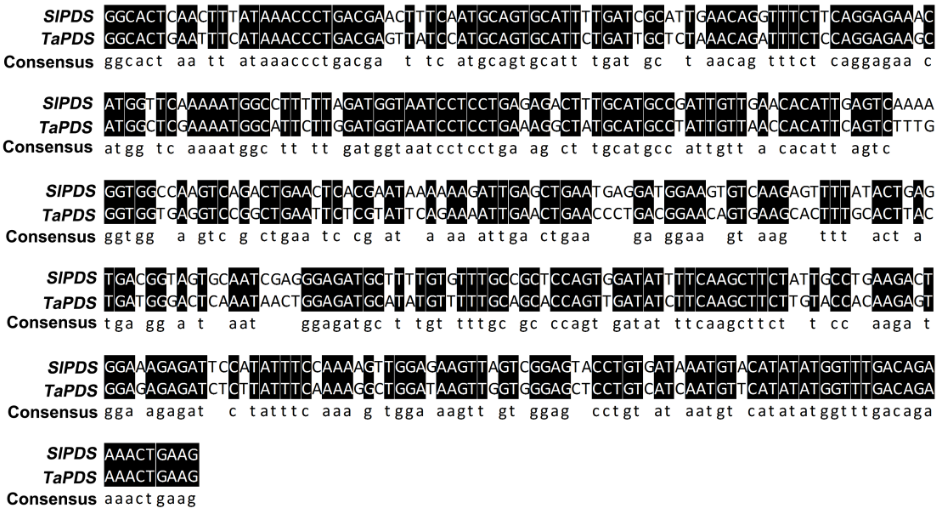

Supplementary Figure S1. Nucleotide sequence alignment of *PDS* fragments from tomato and wheat.

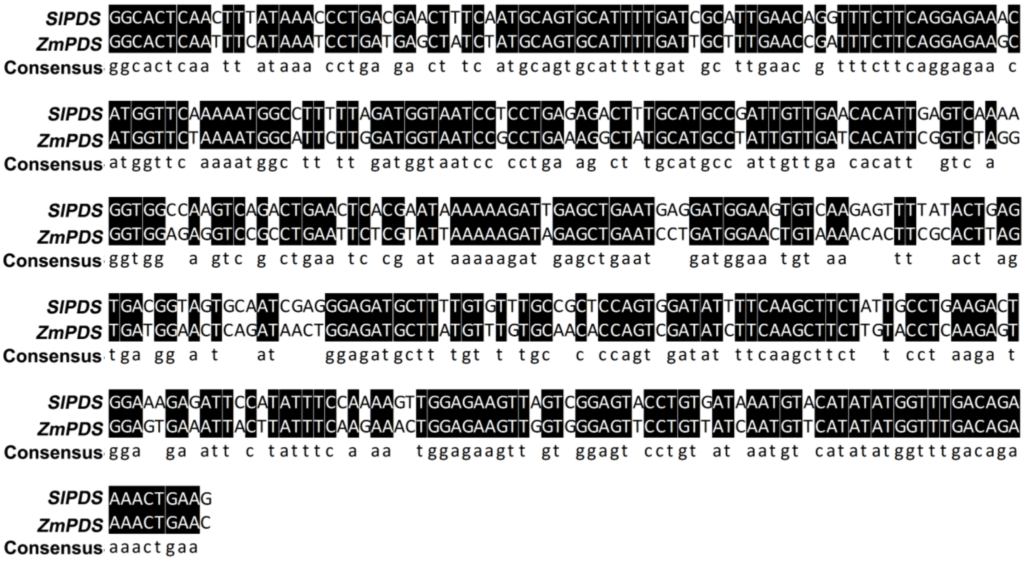

Supplementary Figure S2. Nucleotide sequence alignment of *PDS* fragments from tomato and maize.

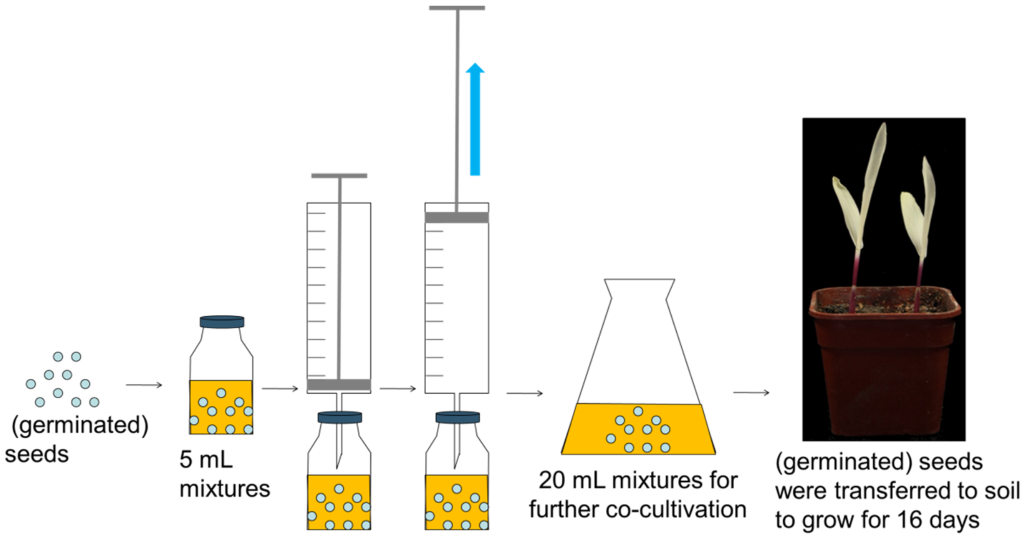

Supplementary Figure S3. Schematic illustration of the vacuum agroinfiltration process. Prepare 20 mL agroinfiltration mixtures, and (germinated) seeds. Place seeds in 5 mL portions of the agroinfiltration mixtures in 10 mL glass bottles with rubber septa, shake and ensure that all seeds are immersed in the mixture. Penetrate the septa with the needle of a 20 mL syringe, and pull the plunger fully into the syringe, thereby creating a vacuum of about 20 kPa for 15 s. Repeat this process from shaking twice (for wheat) or four times (for maize). After co-cultivation (germinated) seeds were transferred to soil to grow for 16 days.

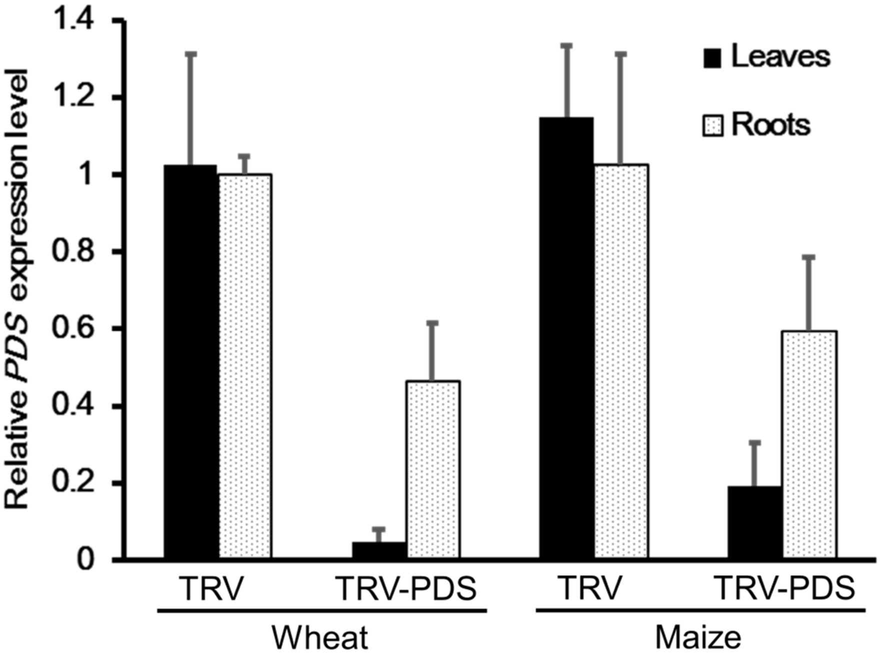

Supplementary Figure S4. Quantitative RT-PCR analysis of TRV-mediated VIGS of *PDS* genes in leaves and roots of wheat and maize. (Using *Actin* as an internal control, repeated more than three times with similar results).

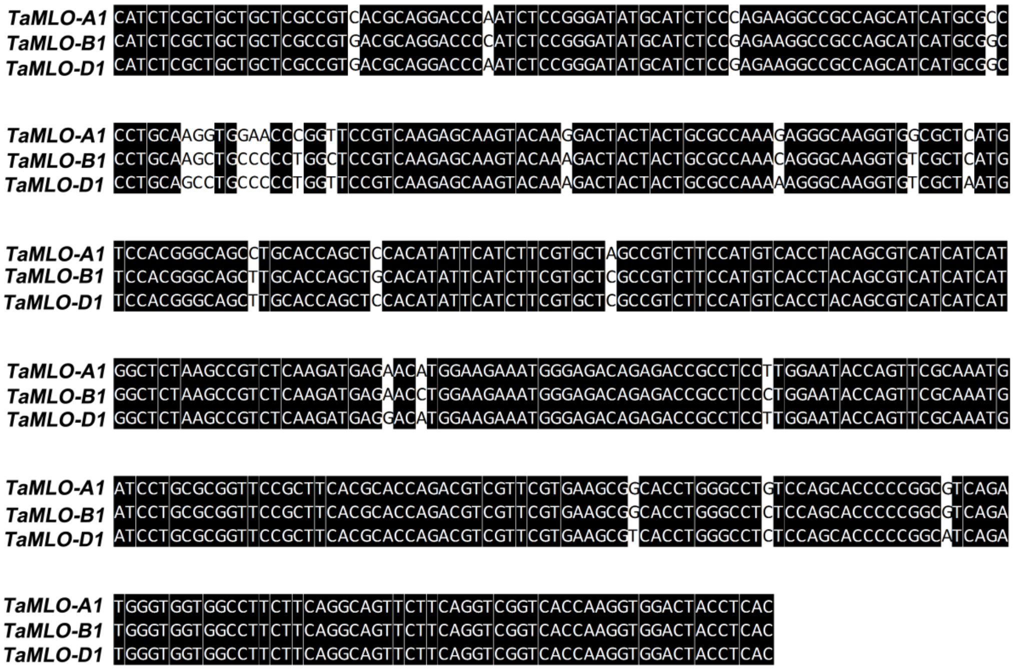

Supplementary Figure S5. Nucleotide sequence alignment of fragments from *MLO* homoeoalleles. *TaMLO-A1*, *TaMLO-B1* and *TaMLO-D1,* located in wheat sub-genomes A, B and D, respectively.

1.2 Supplementary Tables

Supplementary Table S1. Names and sequences of oligonucleotides used in this study.

Name	Sequence
ZJ01	5’-CGGGGTACCGGCACTCAACTTTATAAACC-3’
ZJ02	5’-CGGGGATCCTTCAGTTTTCTGTCAAACC-3’
ZJ03	5’-TATTTGAGTCCCATCAGTAA-3’
ZJ04	5’-ACAGCCGTTTTGATTTCC-3’
ZJ05	5’-GCTTACCTGGCCCGAGAAGGTGAAGTTT-3’
ZJ06	5’-CGGGGATCCTTCAGTTTTCTGTCAAACC-3’
ZJ07	5’-GATACACGCTTCCTCATGCTATCC-3’
ZJ08	5’-AGAGCCACCGATCCAGACACTG-3’
ZJ09	5’-CAATGGCACTGGAATGGT-3’
ZJ10	5’-ATCTTCAGGCGAAACACG-3’
ZJ11	5’-CCGGAATTCCATCTCGCTGCTGCTCGCCG -3’
ZJ12	5’-CGCGGATCCGTGAGGTAGTCCACCTTGGT-3’
